# Aortic and Cardiac Structure and Function Using High-Resolution Echocardiography and Optical Coherence Tomography in a Mouse Model of Marfan Syndrome

**DOI:** 10.1371/journal.pone.0164778

**Published:** 2016-11-08

**Authors:** Ling Lee, Jason Z. Cui, Michelle Cua, Mitra Esfandiarei, Xiaoye Sheng, Winsey Audrey Chui, Michael Haoying Xu, Marinko V. Sarunic, Mirza Faisal Beg, Cornelius van Breemen, George G. S. Sandor, Glen F. Tibbits

**Affiliations:** 1 Child and Family Research Institute, Department of Cardiovascular Sciences, Vancouver, BC, Canada; 2 Department of Biomedical Physiology and Kinesiology, Simon Fraser University, Burnaby, BC, Canada; 3 School of Engineering Science, Simon Fraser University, Burnaby, BC, Canada; 4 Anesthesiology, Pharmacology and Therapeutics, Faculty of Medicine, University of British Columbia, Vancouver, BC, Canada; 5 Division of Cardiology, Department of Pediatrics, UBC, Vancouver, BC, Canada; Scuola Superiore Sant'Anna, ITALY

## Abstract

Marfan syndrome (MFS) is an autosomal-dominant disorder of connective tissue caused by mutations in the fibrillin-1 (FBN1) gene. Mortality is often due to aortic dissection and rupture. We investigated the structural and functional properties of the heart and aorta in a [*Fbn1*^*C1039G/+*^] MFS mouse using high-resolution ultrasound (echo) and optical coherence tomography (OCT). Echo was performed on 6- and 12-month old wild type (WT) and MFS mice (n = 8). In vivo pulse wave velocity (PWV), aortic root diameter, ejection fraction, stroke volume, left ventricular (LV) wall thickness, LV mass and mitral valve early and atrial velocities (E/A) ratio were measured by high resolution echocardiography. OCT was performed on 12-month old WT and MFS fixed mouse hearts to measure ventricular volume and mass. The PWV was significantly increased in 6-mo MFS vs. WT (366.6 ± 19.9 vs. 205.2 ± 18.1 cm/s; *p* = 0.003) and 12-mo MFS vs. WT (459.5 ± 42.3 vs. 205.3 ± 30.3 cm/s; *p*< 0.0001). PWV increased with age in MFS mice only. We also found a significantly enlarged aortic root and decreased E/A ratio in MFS mice compared with WT for both age groups. The [*Fbn1*^*C1039G/+*^] mouse model of MFS replicates many of the anomalies of Marfan patients including significant aortic dilation, central aortic stiffness, LV systolic and diastolic dysfunction. This is the first demonstration of the direct measurement in vivo of pulse wave velocity non-invasively in the aortic arch of MFS mice, a robust measure of aortic stiffness and a critical clinical parameter for the assessment of pathology in the Marfan syndrome.

## Introduction

Marfan syndrome (MFS) is an autosomal-dominant disorder of connective tissue caused by mutations in the fibrillin-1 gene (*FBN1*) located on chromosome 15q15-31 with an estimated prevalence of 1 in 3,000–5,000 [1] leading to multi-systemic clinical problems in the skeletal, ocular and cardiovascular systems; however, sudden death is most often due to thoracic aortic aneurysms leading to aortic dissection and rupture [2, 3]. Fibrillin-1 monomers form complex extracellular microfibrils as scaffolds for elastic fibers in the aorta and other connective tissues [4]. *FBN1* mutations in mice can lead to abnormalities in the structure of the microfibrillar matrix, dysregulation of matrix homeostasis with excess transforming growth factor beta (TGF-β) and matrix metalloproteinases (MMPs) expression and abnormal cell-matrix interactions in the heart and aorta [5–8]. Previously, we demonstrated that mutations in *FBN1* in MFS mice resulted in fragmentation and disorganization of elastin fibers, with significant detrimental effects on vascular smooth muscle and endothelial cells function within the aortic walls [8–12]. However, it is not clear whether the pathological processes seen in the aorta also affect cardiac function in MFS mice and patients.

Marfan cardiovascular manifestations include mitral valve prolapse, mitral annular calcification, ascending and descending aortic dilatation and dissection, aortic valvular regurgitation, and dilated cardiomyopathy which can all be monitored by various modes of echocardiography [13–16]. In addition to evaluating the structural and functional properties of the heart and aorta, pulse wave Doppler measurements of pulse wave velocity (PWV) can estimate aortic stiffness [17].

Crucial to our understanding of the role of *FBN1* mutations in the etiology of MFS has been the development of mouse models in which the fibrillin-1 gene has been modified. The first MFS mouse model created was the homozygous *mgΔ/mgΔ* mouse in which exons 19 to 24 were replaced with the neomycin (neo) gene under the control of the PGK promoter to mimic the dominant-negative effect of fibrillin-1 mutations seen in some MFS patients [18]. While the heterozygote animals were histologically indistinguishable from wild-type mice, the homozygote mice died before the second week of life, due to cardiovascular failure. Expression analysis showed that the *mgΔ* allele had a 90% lower transcript level compared to the normal *FBN1* allele. Although the *mgΔ/mgΔ* mice were instrumental in identifying an important function of fibrillin-1 in MFS, their early demise hampered the characterization of the precise pathogenic sequence leading to an aortic aneurysm. Subsequently a *mgR* homozygote MFS mouse model was created by inserting a PGK neo-cassette between exons 18 and 19 of the endogenous gene [19]. This results in a hypomorphic mutation, and *Fbn1*^*mgR/mgR*^ mice exhibit a ~5-fold reduction in the expression of the *mgR* allele, which produces low levels of normal fibrillin-1 and homozygous *mgR* mice which typically die at ~4 months of age [19].

A third MFS mouse model (*Fbn1*^*C1039G/+*^), used in the present study, is created by a heterozygous substitution of a cysteine by a glycine at residue 1039, in an epidermal growth factor (EGF) domain of fibrillin-1, which is considered as one of the most common types of MFS mutations in humans [20]. These heterozygote mice exhibit impaired microfibrillar deposition, skeletal deformity, and progressive deterioration of aortic wall architecture, recapitulating the human condition [20]. These data are consistent with a model that invokes haploinsufficiency for wild-type fibrillin-1, rather than production of mutant protein, as the primary determinant of failed microfibrillar assembly. The haploinsufficiency results in ~½ the normal fibrillin concentration which directly contributes to the progression of an aortic aneurysm. These mice typically develop advanced aneurysms by 9 months, but early signs of elastin fragmentation are observed around 3 months of age [11]. These mice die typically from an aortic aneurysm between 12–18 months [20]. In this study we used the *Fbn1*^*C1039G/+*^ MFS mouse model because the more natural progression of the disease allowed us to examine the time course of the pathological phenotype over a 12 month period.

While both the *Fbn1*^*mgR/mgR*^ and the *Fbn1*^*C1039G/+*^ MFS mouse models continue to reveal tremendous insight into the impact and consequences of *FBN1* mutations in the etiology of Marfan syndrome, there have not been any *in vivo* non-invasive echo studies of the clinical phenotype of these mice to determine the degree to which they recapitulate the human condition [20]. The goal of this study was to investigate the structural and functional properties of the heart and aorta, particularly pulse wave velocity and other well characterized indices known to be abnormal in humans with MFS, in the *Fbn1*^*C1039G/+*^ mice using both high-resolution ultrasound and optical coherence tomography. Moreover, an important advantage of the echo Doppler method in the *Fbn1*^*C1039G/+*^ mouse is that it allows for evaluation of the disease progression in the same animal longitudinally.

## Materials and Methods

### Animal care and preparation

Six- and twelve-month old MFS mice that were heterozygous for an *Fbn1* allele (*Fbn1*^C1039G/+^) and wild-type (WT) littermates (*Fbn1*^+/+^) were studied. *Fbn1*^C1039G/+^ mice were mated with WT mice to generate *Fbn1*^C1039G/+^ and *Fbn1*^+/+^ littermates. Animals were housed in the animal facility of the Child and Family Research Institute (CFRI) with standard animal room conditions (25°C, 12-hour light-dark, ≤5 animals in a cage). All experimental procedures were approved by the University of British Columbia animal ethics board and were in accordance with the Canadian Council on Animal Care (CCAC) guidelines.

### Echocardiography

A Vevo 2100 ultrasound system (VisualSonics, Toronto, ON, Canada) equipped with a MS550 transducer was used for mouse echocardiography. The transducer had a central frequency of 40 MHz, a focal length of 7.0 mm, and a frame rate of 557 fps (single zone, 5.08 mm width, B-mode). The maximum field of view of two-dimensional (2D) imaging was 14.1 x 15.0 mm with a spatial resolution of 90 μm (lateral) by 40 μm (axial).

Anesthesia was induced by putting the mouse in an induction chamber using 3% isoflurane and 1 L/min 100% oxygen for 1–2 minutes. Once the animal lost its righting reflex, it was laid supine on a heated platform with its nose enveloped in a nosecone to keep the mouse anesthetized by 1.5–2% isoflurane [21, 22]. The mouse limbs were taped to four electrocardiogram (ECG) electrodes which were imbedded in the platform for the measurement of heart rate, ECG and respiratory rate. Body temperature was monitored through a rectal probe, and maintained at 36–38°C with a heating lamp and a heated platform.

**Fig 1:**
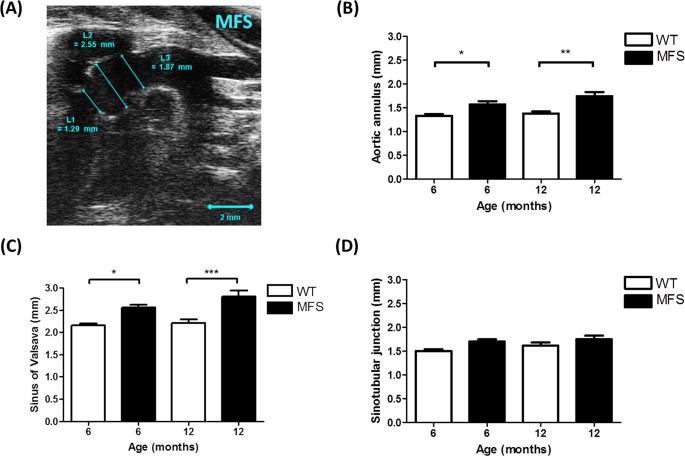
Aortic root dimension of WT and MFS mice. **(A)** B-mode view of the aortic arch from a 6-month MFS mouse. Diameters of the **(B)** aortic annulus [L1], **(C)** sinus of Valsalva [L2] and **(D)** sinotubular junction [L3] were significantly increased in MFS mice versus WT. The larger aortic root diameter indicates significant aortic dilation. * indicates *p* < 0.05, ** indicates *p*< 0.01, *** indicates *p*< 0.001. (n = 8)

**Fig 2:**
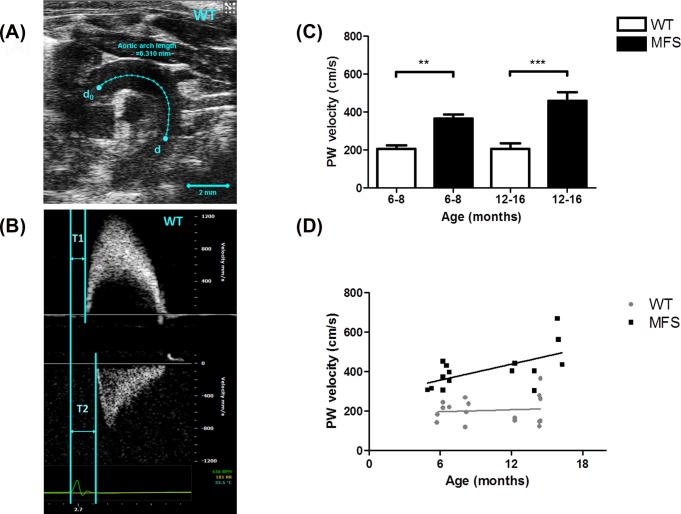
Pulse wave velocity (PWV) of aortic arch. **(A)** B-mode view of aortic arch of a 6-month wild type (WT) mouse. The distance between the ascending and descending aorta pulse wave Doppler recordings is indicated by the blue line (d_0_ to d). Scale bars, 2 mm. **(B)** Pulse wave Doppler tracing of the ascending (upper panel) and descending aorta (lower panel). The X-axis represents time (ms) and Y-axis represents blood flow velocity (mm/s). T1 is measured from the beginning of the QRS wave on the ECG to the beginning of the ascending aortic peak velocity and T2 is the beginning of the QRS wave on the ECG to the beginning of the descending aortic peak velocity. Pulse wave velocity was calculated using the distance between d_0_ and d in the aortic arch divided by the transit time (i.e. [d-d_0_] / [T2-T1]). **(C)** Aortic PWV of WT and MFS mice from two age groups (6–8 months and 12–16 months group). PWV was significantly increased in 6–8 months MFS mice compared to WT (*p* = 0.003) and in 12–16 months MFS mice compared to WT (*p*< 0.001), respectively. **(D)** Correlations between age (x-axis) and PWV (y-axis) of WT (●) and MFS (■) mice. PWV in MFS mice was directly proportional to age (R-squared = 0.356, *p* = 0.02), but not in WT mice. ** indicates *p*< 0.01, *** indicates *p*< 0.001. (n = 8)

Aortic diameters (aortic annulus [L1], sinuses of Valsalva [L2] and sinotubular junctions [L3]) were measured from the B-mode aortic arch view (Fig 1A). The ascending and descending aortic peak velocities were measured from the pulse wave (PW) Doppler-mode aortic arch view. Pulse wave velocity (PWV) was obtained from the B-mode and Doppler-mode aortic arch view, calculated as PWV = aortic arch distance / transit time (cm·s^-1^) [17]. The PW Doppler mode sample volume was placed in the ascending aorta and the time (T1) from the onset of the QRS complex to the onset of the ascending aortic Doppler waveform was measured (Fig 2A). On the same image plane, the PW Doppler mode sample volume was placed as distal as possible in the descending aorta and the time (T2) from the onset of the QRS complex to the onset of the ascending aortic Doppler waveform was measured. Obtained values for T1 and T2 were averaged over 10 cardiac cycles. The aortic arch distance was measured between the 2 sample volume positions along the central axis of aortic arch on the B-mode image, and the transit time was calculated by T2 –T1 (ms) [17].

The left ventricular (LV) structural and functional parameters were calculated from the LV parasternal short-axis M-mode view, which was recorded at the level of two papillary muscles. An M-mode cursor was positioned perpendicular to the anterior and posterior walls, in the middle of the LV for measuring wall thickness and chamber dimensions. LV functional parameters [stroke volume (SV), ejection fraction (EF), fractional shortening (FS) and cardiac output (CO)] and structural parameters [end-systolic volume (ESV), end-diastolic volume (EDV), LV mass, anterior and posterior wall thickness)] were obtained from this M-mode view. Interventricular septal wall was measured from LV parasternal long-axis M-mode view. Values for LV structural parameters were normalized by body weight to avoid individual differences. Mitral flow velocity including both early (E) and atrial (A) velocities were acquired from the Doppler-mode apical four chamber view. The isovolumic contraction time (IVCT), isovolumic relaxation time (IVRT) and ejection time (ET) were also measured from this view [23]. The myocardial performance index (MPI), calculated by [(IVCT + IVRT)/ ET], was used for evaluating LV systolic function [24].

### Histological imaging of the aorta

For the histological analysis, mice (n = 3) were anaesthetized with 3% isoflurane. After the pedal reflex was lost, mice were sacrificed by cervical dislocation. The aortas were dissected from the animals and fixed in 10% buffered formalin for 48 hours. Later they were immersed in 70% ethanol overnight at 4°C, and then embedded in paraffin [11]. Tissue specimens were transversely sectioned at 5 μm and deparaffinized in xylene and rehydrated in graded ethanol. Elastic and collagen fibers of aortas were stained by use of Accustain® Elastic Stain kit (Sigma-Aldrich, St. Louis, MO) according to the manufacturer’s instructions and as described previously [11]. The number and percentage area of elastic fibers in the aorta were determined by using Amira software (FEI, France), a commercial three-dimensional (3D) image processing and analysis program. By tracing the elastic fibers for the whole transverse section, pixel size was counted and then converted to the actual size. MATLAB (Mathworks, Natick, MA) was used for calculating the area of elastic fibers of the aorta.

### Optical coherence tomography

Optical coherence tomography (OCT) is an imaging modality that complements ultrasound to investigate cardiac morphological changes. OCT provides depth-resolved, cross-sectional volumes at high spatial resolution (~10 μm) but limited penetration depth (1–2 mm) in cardiac tissue [25]. OCT is similar in principle to ultrasound, except that it uses the transit time of light rather than sound [26]. The volumetric data provided by OCT can be used to quantify structural parameters of the excised mouse hearts, such as wall thickness, wall masses, and luminal volumes. A custom-designed swept-source optical coherence tomography (SS-OCT) system for cardiac imaging was used to image the fixed hearts. Twelve-month-old MFS (n = 8) and WT mice (n = 5) were studied. In preparation for extracting the heart, the mice were anesthetized via an intraperitoneal injection of 40 μL Somnotol (32.5 mg/ml at 65 mg/kg) and heparin (100 μL, at 1000 U/mL and 5000 U/kg). After loss of the mouse toe pinch response, the hearts were excised and then cannulated at the aorta on a Langendorff apparatus and perfused with Tyrode’s solution (4 min at 2 mL/min) and 4% paraformaldehyde (30 min at 2 mL/min) to remove the blood and fix the tissue. Hearts were stored at 4°C in PBS and immersed in glycerol (50% and 70% for 1 day each) before imaging.

The hearts were mounted onto the imaging system using a Luer adapter that was connected to the aortic cannula. The system had an axial resolution of 7 μm and a lateral resolution of 15 μm. The acquired tomographic volumes had a voxel size of 20 x 20 x 20 μm^3^. The OCT volumes of the mouse heart were semi-automatically segmented using Amira software. The ventricular tissue and lumen were segmented and then quantified by considering the number of assigned voxels and the size of each voxel [27]. For the ventricular mass, the specific gravity of myocardium was taken to be 1.055 g·cm^-3^. The 3D OCT data was also used to investigate the validity of the estimation algorithm used by the Vevo 2100 ultrasound system for different cardiac conditions. The lumen diameter was measured from the 3D OCT data and used to estimate the LV volume using the Teichholz equation [28]. For each heart, a trained small-animal sonographer selected the short-axis slice in the OCT volume that best corresponded to the mid-papillary level slice as observed in echocardiography. The short-axis slice was oriented perpendicular to the long axis of the LV, which was estimated by modelling the LV as an ellipsoid. The maximum diameter of the lumen was automatically determined at a constrained angle, from anterior to posterior wall, to match the echocardiographic measurement. The estimate of the volume was then compared to the value computed directly from the 3D OCT data by counting the voxels contained within the semi-automatically segmented lumen. Details of the image acquisition and processing protocols are described in a previous publication [27].

### Statistics

For the echocardiography analysis, all measurements were produced using the VisualSonics cardiac-package software and the measurement of each parameter was repeated over five cardiac cycles to reduce possible bias. All image acquisitions and analyses were conducted by a single investigator who was blinded as to animal genotypes. Statistical analysis was performed using two-way analysis of variance (ANOVA) test followed by Tukey HSD test (JMP version 12). A two tailed *p* value less than 0.05 was accepted as statistically significant. Ordinary least-squares regression analysis was used to test relationships between PWV and mouse age.

For histological quantitative and statistical analysis, two-way ANOVA followed by Tukey HSD test (JMP version 12) was used to compare of parameters among the four groups, 6-month and 12-month, MFS and WT mice with a *p* value < 0.05 being considered significant.

All of the OCT acquisitions and analyses were conducted in a blinded fashion with respect to the genotype of the animals from which each heart was obtained. All manual measurements were acquired three times to determine variability. Independent Student’s t-test (JMP version 12) was used to determine statistical significance between the 12-month-old WT and MFS groups, with *p*<0.05 as the threshold. Pearson’s product-moment correlation coefficient was used to establish strength of correlation in the relationship between the estimated LV volume and the actual LV volume, for both the WT and MFS groups. Data points that were more than two standard errors of the mean (SEM) away from the mean value, calculated with the potential outlier excluded, were taken as outliers. Hearts that had two of more outlying data points were excluded from the statistical analyses.

## Results

### Echocardiography

There were no significant differences in heart rate (HR), body weight (BW), EF and FS between WT and MFS mice groups at both 6 and 12 months of age (Table 1). The aortic annulus diameter was significantly increased by 18% in MFS versus WT in the 6-month (*p* = 0.046) and by 27% in the 12-month (*p* = 0.001) groups, respectively (Fig 1).

**Table 1:** Echocardiographic functional analysis for WT and MFS mice.

Parameter (units)	6 month group			12 month group		
	**WT**	**MFS**	***p***	**WT**	**MFS**	***p***
Body weight (g)	**39.1 ± 1.0**	**37.6 ± 1.1**	**0.88**	**41.9 ± 1.3**	**37.2 ± 2.2**	**0.14**
Heart Rate (bpm)	**434 ± 21**	**432 ± 10**	**0.99**	**453 ± 10**	**462 ± 18**	**0.97**
ESV (N) (μl/g)	**0.76 ± 0.08**	**0.81 ± 0.08**	**0.98**	**0.78 ± 0.07**	**0.62 ± 0.11**	**0.41**
EDV (N) (μl/g)	**1.96 ± 0.09**	**2.37 ± 0.14**	**0.13**	**1.97 ± 0.09**	**1.90 ± 0.17**	**0.98**
SV (N) (μl/g)	**1.20 ± 0.05**	**1.56 ± 0.09**	0.04*	**1.20 ± 0.07**	**1.29 ± 0.13**	**0.88**
EF (%)	**62 ± 3**	**66 ± 2**	**0.75**	**61 ± 2**	**68 ± 3**	**0.33**
FS (%)	**33 ± 2**	**36 ± 1**	**0.74**	**32 ± 1**	**38 ± 2**	**0.29**
CO (N) (ml/min/g)	**0.52 ± 0.03**	**0.68 ± 0.05**	**0.09**	**0.54 ± 0.03**	**0.60 ± 0.06**	**0.81**

All parameter measurements are presented as Means ± SEM (n = 8 mice).

* indicates *p* < 0.05.

N, normalized with body weight. ESV End systolic volume; EDV End diastolic volume; SV Stroke volume; EF Ejection fraction; FS Fractional shortening; CO cardiac output.

The diameter of the sinus of Valsalva was also significantly increased in MFS mice versus WT by 19% in 6-month (p = 0.01) and by 27% in 12-month (p< 0.001) groups, respectively (Fig 1). PWV was increased by 79% in 6–8 month old MFS mice compared to WT (*p* = 0.003) and by 124% in 12–16 month MFS mice compared to WT (*p*< 0.001). The relationship between age (x-axis) and PWV (y-axis) of MFS and WT mice is shown in Fig 2. PWV in MFS mice increased linearly with age (R-squared = 0.356, *p* = 0.02), but not in WT mice (R-squared = 0.008, *p* = 0.73). The estimated regression lines are y = 0.45x + 275.49 in the MFS group and y = 0.06x + 187.01 in the WT group.

**Fig 3:**
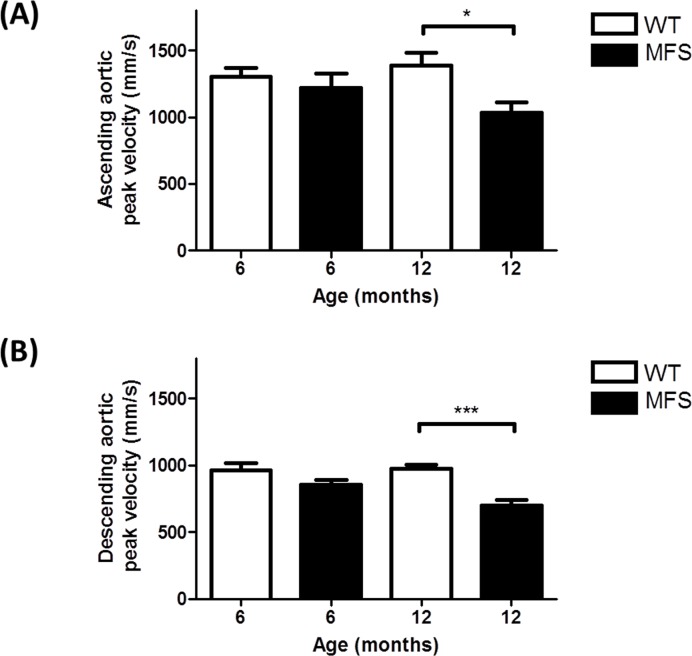
Peak velocity was calculated from pulsed-wave Doppler mode from an aortic arch view. **(A)** Ascending aortic peak velocity of WT and MFS mice from two age groups (6- and 12-month). Ascending aortic peak velocity was significantly decreased in 12-month MFS mice versus WT mice (*p* = 0.04). **(B)** Descending aortic peak velocity was significantly decreased in 12-month MFS mice (*p*< 0.001) versus WT mice. * indicates *p*< 0.05, *** indicates *p*< 0.001. (n = 8)

Fig 3 shows that the ascending aortic peak velocities were significantly decreased by 25% (*p* = 0.04) in 12-month MFS versus WT mice. Descending aortic peak velocity was decreased by 28% in 12-month MFS versus WT mice (*p*< 0.001) and by 18% in 12-month versus 6-month MFS mice (*p* = 0.01) (Fig 3).

**Fig 4:**
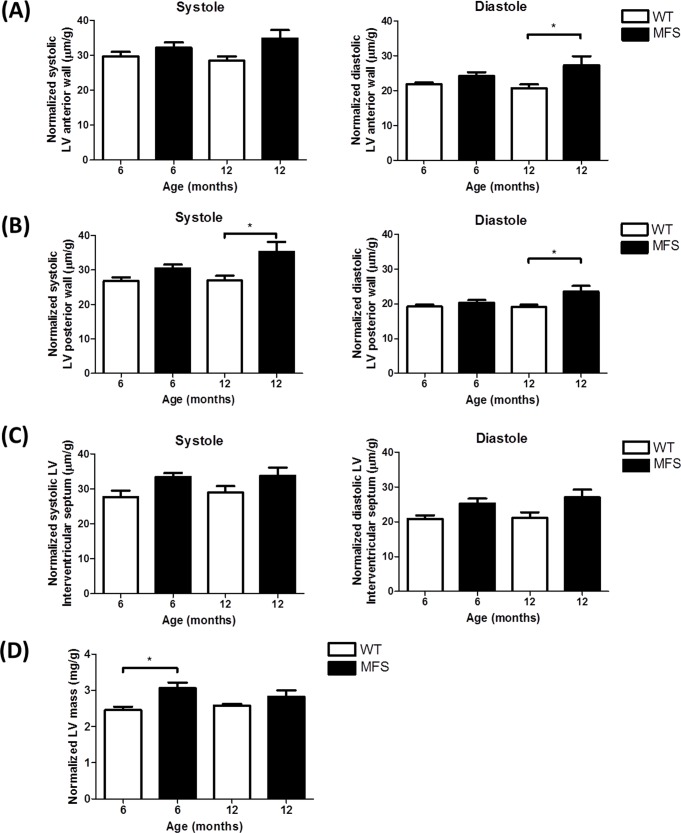
Echocardiographic assessment of left ventricle (LV) mass and wall thickness. Left ventricle (LV) mass, systolic and diastolic wall thickness were normalized by body weight (g). Data presented are normalized systolic and diastolic **(A)** anterior wall thickness, **(B)** posterior wall thickness, **(C)** interventricular septal thickness and **(D)** normalized LV mass from WT and MFS mice (n = 8). * indicates *p*< 0.05.

Normalized diastolic LV anterior wall thicknesses was significantly increased by 32% in MFS mice compared to WT at 12-months of age (p = 0.03) (Fig 4). Normalized systolic (*p* = 0.01) and diastolic (*p* = 0.02) posterior wall thickness were also increased by 31% and 23% in MFS mice compared to WT in the 12-month group. The normalized LV mass which was calculated from these measurements, was significantly increased by 25% in MFS mice compared to WT at 6-months of age (*p* = 0.01) (Fig 4).

**Fig 5:**
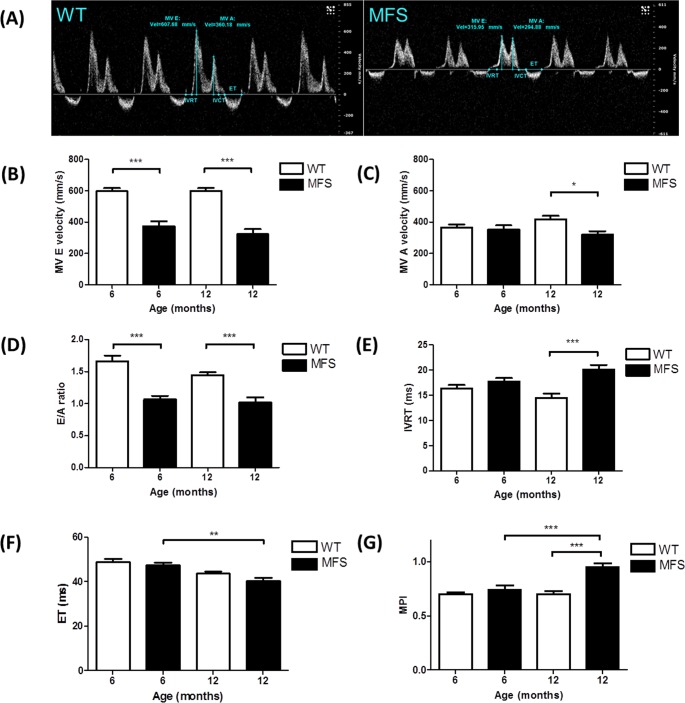
Mitral inflow velocity of WT and MFS mice. **(A)** Mitral inflow velocity profile of WT (left panel) and MFS (right panel) mice at 6 months. Velocity (mm/s, y-axis) is shown as a function of time (ms, x-axis). Mitral valve early peak (MV E) velocity was determined from the first peak and atrial peak velocity (MV A) from the second peak. Isovolumic relaxation time (IVRT), isovolumic contraction time (IVCT) and ejection time (ET) are displayed in blue and red lines, respectively. **(B)** MV E velocity, **(C)** MV A velocity, **(D)** E/A ratio, **(E)** IVRT, **(F)** ET and **(G)** Myocardial performance index (MPI) were measured from WT and MFS mice (n = 8). * indicates *p*< 0.05, ** indicates *p*< 0.01, *** indicates *p*< 0.001.

Mitral valve E velocity was decreased by 37% and 46% in MFS mice compared to WT at both 6-months (*p*<0.001) and 12-months of age (*p*<0.001), respectively (Fig 5). Mitral valve A velocity showed no difference between MFS and WT mice at 6-months, but was decreased significantly by 24% in MFS at 12-months (*p* = 0.03). As a consequence, the E/A ratio was significantly decreased in MFS mice versus WT in both 6-month (36%, *p*< 0.001) and 12-month (29%, *p*< 0.001) groups, respectively (Fig 5). IVRT was significantly prolonged in 12-month old MFS mice compared with WT (*p*< 0.001). There was no difference in EF between MFS and WT mice at 6- and 12-months, but EF was significantly decreased in the 12-month old MFS group compared with 6-month old MFS group (*p* = 0.003). The MPI was increased by 36% in 12-month old MFS mice (*p*< 0.001) compared with WT and was increased by 28% in 12-month old MFS mice (*p*< 0.001) in contrast to 6-month MFS mice (Fig 5).

**Fig 6:**
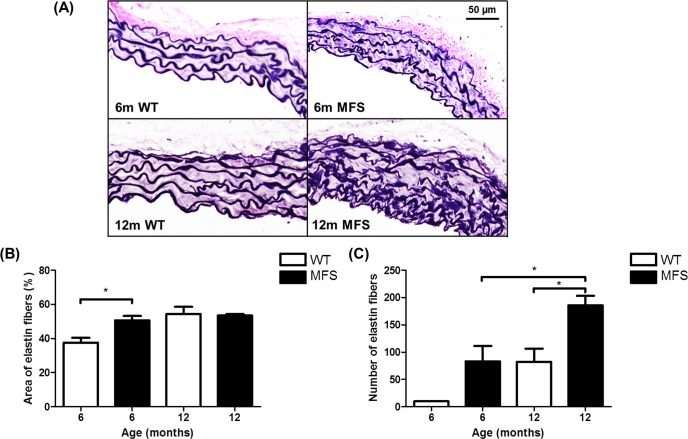
Histological analysis of WT and MFS mice aorta. **(A)** Representative histological images stained with Van Gieson's staining reveal the arrangement of elastin (dark blue) and collagen (pink) in the cross-section of the aorta from 6-month and 12-month wild-type (WT) and Marfan (MFS) mice. Elastin fibers display severe fragmentation and disorganization in the MFS aorta as compared with WT samples. **(B)** The area and **(C)** number of elastin fibers were measured by tracing the elastin fibers on the transverse section of mouse aorta. * indicates p< 0.05 (n = 3).

Histological imaging of the aortas from 6 month and 12 month WT and MFS showed the loss of organization and disruption of the elastin fibers as illustrated in Fig 6. With Van Gieson’s staining, elastin is illustrated in dark purple, whereas collagen is present in light pink. Elastin fibers fragmentation is visible in the MFS aorta compare to WT. The area of elastin fibers was significantly increased in MFS mice versus WT in 6-month of age (26%, *p* = 0.02). The number of elastin fibers was significantly increased in 12-month MFS mice versus 12-month WT (125%, *p* = 0.03) and versus 6-month MFS mice (123%, *p* = 0.03).

### Optical coherence tomography

**Fig 7:**
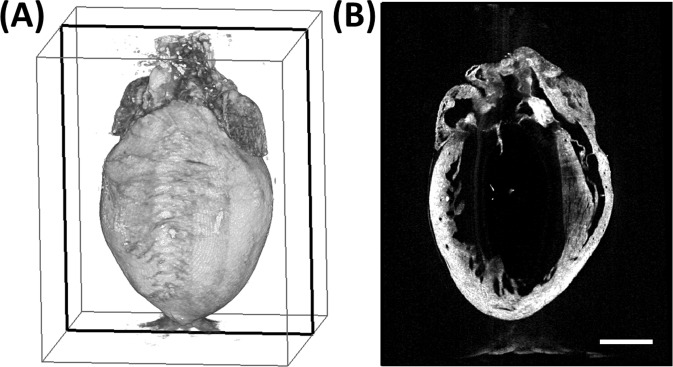
Representative 3D high-resolution volumetric OCT data set. **(A)** Volumetric rendering of representative OCT data set acquired from a MFS mouse. **(B)** Cross-sectional view at the plane indicated by the black box in (A). Scale bar denotes 2 mm.

A representative OCT data set is displayed in Fig 7. The structural parameters obtained from the OCT data are displayed in Table 2. The ventricular masses and luminal volumes were normalized to body weight. There were no significant differences in normalized heart weight and ventricular mass between the 12-month WT and MFS groups. The body weight was decreased by 16% in MFS mice (*p*< 0.05), while the left ventricular volume was increased by 30% (*p*< 0.05). Although no differences were found in the normalized ventricular mass, the LV mass-to-volume ratio was decreased by 19% (*p*< 0.01).

**Table 2:** Structural properties of 12-month-old WT and MFS Mice in OCT study.

Parameters (unit)	WT (n = 4)	MFS (n = 7)	P
**BW (g)**	45.90 ± 0.84	38.57 ± 1.96	0.025*
**HW (mg/g)**	5.72 ± 0.65	6.18 ± 0.41	0.574
**LV Volume (μL/g)**	2.22 ± 0.18	2.87 ± 0.16	0.029*
**LV Mass (mg/g)**	2.86 ± 0.16	3.02 ± 0.15	0.490
**LV Mass/Vol (mg/μL)**	1.30 ± 0.04	1.06 ± 0.04	0.003**

All results are presented as means ± SEM.

* indicates *p*< 0.05

** indicates *p*< 0.01.

**Fig 8:**
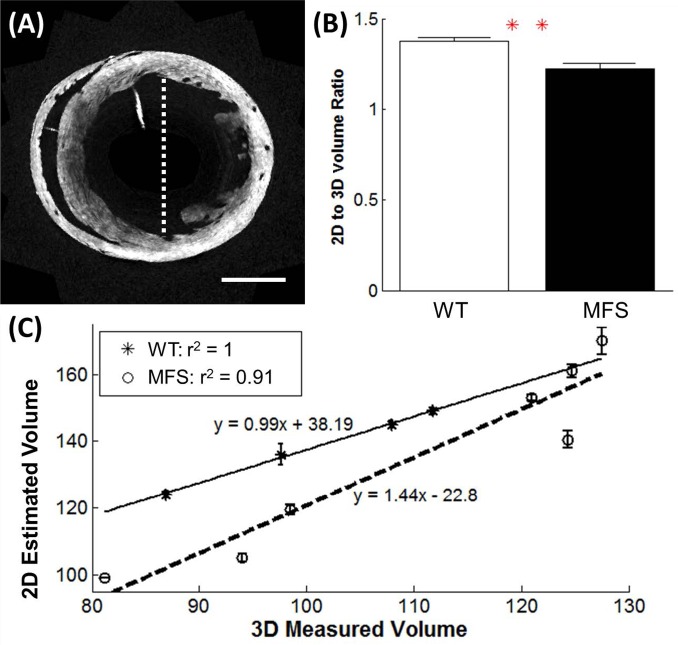
Comparison of the 2D and 3D LV volume measurements from the OCT data set. Comparison of the LV volume, as determined by the Teichholz method based on 2D measurements on the OCT data, and by direct volume measurement from the 3D OCT data set. (A) The diameter of the lumen was measured from the short-axis slice located at the mid-papillary level, from the anterior to posterior wall, as shown by the dashed white line. The volume was estimated from the diameter measurement using Teicholz’s equation. Scale bar denotes 2 mm. (B) The ratio of the estimated volume (using Teichholz method) to measured volume (computed in 3D) was significantly different between the WT and MFS mice (*p*< 0.01). (C) Linear regression on the estimated volume (in 2D) and actual volume (computed in 3D). The LV volumes of the MFS mice are overestimated in the 2D estimation formula by up to 40% (WT, n = 4; MFS, n = 7). ** indicates *p*< 0.01.

For the measurement of LV volume, there was a strong linear correlation between the value estimated from the 2D diameter measurement, and the directly measured 3D parameters in both the 12-month WT and MSF groups (r = 1 and r = 0.95, respectively) (Fig 8). However, there was a significant difference between the MFS and WT mice for the ratio of the estimated and measured volume (*p*< 0.01).

### Discussion

Aortic root aneurysm is the most prominent and life-threatening feature of MFS cardiovascular complications [29]. Increased aortic stiffness and loss of wall elasticity are considered as important detrimental factors contributing to aneurysm progression. Early studies in mouse models of MFS have used *ex-vivo* approaches to indirectly measure aortic wall stiffness/elasticity including the use of isometric wire myography [8]. Later, non-invasive high resolution high frequency ultrasound imaging techniques provided a more powerful tool for in vivo measurements of both cardiac and aortic function and structure in real time. This is the first *in vivo* mouse study to duplicate studies in MFS patients showing dilation of the sinuses of Valsalva, sino-tubular junction and progressive loss of aortic root elasticity [30–32] using a non-invasive and high resolution ultrasound technique. As one would predict, as the vessel diameter increased, the peak blood flow velocity decreased [33]. Our study also showed a significantly decreased peak velocity in both the ascending and descending aortas. These results were more pronounced in the 12-month group, which is consistent with progressive dilation in the aorta of MFS mice with aging.

Even without aortic root dilation, MFS patients have been shown to have increased aortic stiffness [17]. PWV has been reported to be a robust marker of aortic stiffness and can be measured non-invasively using echocardiography in patients [32]. PWV has been demonstrated in different populations including elderly, hypertensive, diabetic and renal patients as an index of aortic stiffness and the earliest predictor of cardiovascular risk [34–37]. Using echocardiography or magnetic resonance imaging (MRI) previous studies have confirmed increased aortic stiffness in MFS [38–41]. In patients with MFS, it has been demonstrated that aortic stiffness was increased with age and aortic diameter [42]. Our echocardiographic Doppler method of assessment of the biophysical properties of the aorta in mice was based on that created by Bradley et al [17] for use in humans and who reported an increased PWV in pediatric patients with MFS compared with normal subjects (481 ± 70 vs. 357 ± 61 cm/s, *p*< 0.001). This PWV method also has been shown by Kiotsekoglou et al. to be a reliable indirect aortic stiffness measurement in MFS patients. Our data are also consistent with previous human studies using PWV analysis methods on MFS patients [17, 39], and therefore, confirming for the first time that PWV measurement using 2D and Doppler echocardiography as performed in this study can be reliably applied to the mouse for evaluating aortic stiffness. Similar observations of increased aortic stiffness were made by our group in the *ex vivo* MFS mouse model using wire myography [8].

Different methods of measuring PWV have been reported in mice. They also differ in what they measure, some measure the velocity of the flow wave, others determine the velocity of the pressure wave in the aortic wall and others a combination of both. Hartley et al. (1997) calculated upper abdominal aortic PWV in mice by dividing the fixed distance from the aortic arch to the abdominal aorta 4 cm downstream by the pulse transit time which they measured using the Doppler waveform synchronized with ECG [43]. PWV has also been measured by using a flow/area (QA) method involving aortic Doppler flow and aortic area change [44, 45]. However, the QA method is complex as it requires angle correction of the Doppler velocity and needs careful imaging of the aorta. Rabben et al. (2004) compared the QA method with the Bramwell–Hill (BH) method and concluded that the QA method needed refining [46]. Another study applied carotid-femoral applanation tonometry which is a commonly used method clinically for measuring PWV in mice [47]. In comparison, our method is relatively simple and direct, without the need for simultaneous blood pressure measurements or assumptions about Doppler angle to obtain flow volumes. Furthermore, the technique presented in this study focuses on the localized distance of aortic root which is the most important locus for the MFS characterization. It has the potential to be repeated for long term longitudinal in vivo studies.

In our study, PWV was significantly increased in 6–8 and 12–16 month old MFS mice compared with WT and increased linearly with age. These data indicate a continuing process of aortic stiffening in MFS mice with aging. This may increase the LV afterload, potentially leading to cardiac remodeling.

The difficulties of this method in mice include the extreme shortness of the aortic arch (~5.5 mm) and the very rapid transit time due to the high heart rate. However, with repeated measurements over ten cardiac cycles, the accuracy can be excellent making this a robust technique for the evaluation of aortic stiffness in the mouse.

The etiology for the increased PWV in MFS is likely based on a loss of aortic elastin fibril organization and integrity. In this study this was clearly demonstrated in both the 6 month old and 12 month old groups using histology in the fixed aortic samples (Fig 6). A more detailed examination of this approach can be seen in our previous study [11] the basic results of which are corroborated in the present study. Recently an MRI-based study was performed with LMI1174—a gadolinium-based elastin-specific magnetic resonance contrast agent (ESMA) which accurately measures elastin bound gadolinium within the aortic wall [48]. In this study they found that in the 8 month *Fbn1*^C1039G/+^ MFS mouse, there was a significant decrease in aortic wall elastin compared to WT.

Both echocardiography and OCT showed no difference in normalized LV mass between the 12–month WT and MFS mice. For the LV volume, the echocardiography results showed no significant difference in either the end systolic or end diastolic volumes. However, with the OCT results, the MFS mice showed significantly larger LV volumes. The regression analyses indicate that the Teichholz estimation of the LV volume overestimates that for MFS mice in a manner which increases as a function of measured volume relative to the WT groups. This suggests that the estimation method used in echocardiography may not be as sensitive as other methods for measuring LV volume [27]. Cardiomyopathy could alter the shape of the LV in the MFS mice, thus, using a formula for LV volume calculation from 2D measurements is less accurate for comparison between different conditions, since the formula assumes normal LV geometry. However, for both the MFS and WT mice, there was a strong linear correlation between the estimated (2D) and the actual volume (3D), which suggests that the estimation method can be used to compare accurately the LV volume within a single sub-type.

The MPI data suggests that MFS mouse hearts may have some degree of systolic dysfunction and the Doppler mitral flow data suggests the equivalent of type 1 diastolic dysfunction, i.e. abnormal myocardial relaxation. There was a tendency towards cardiac hypertrophy which is consistent with previous clinical findings [49, 50]. The question remains whether this is a primary ventricular effect or secondary to aortic dysfunction or a combination of both. While fibrillin-1 is mainly expressed in the proximal aorta, it is also present in the myocardium, albeit to a lesser degree. Therefore, MFS media degeneration and aortic aneurysm are most prevalent in the ascending aorta [51]. In MFS mice, the increased aortic stiffness could lead to greater afterload, resulting in an increase in LV work [30]. Consequently, it is possible that the LV wall thickness is increased in MFS mice as a compensatory mechanism to decrease wall stress. However, some studies in both human and murine models [52–55] are suggestive of a primary cardiomyopathy in MFS but this remains controversial. Abnormal LV elastic recoil, presenting early in life suggests an intrinsic abnormality of the myocardium [56]. Previous data suggested the presence of LV diastolic dysfunction in young MFS patients, who showed a significant increase in left ventricular end-diastolic diameter and isovolumic relaxation time (IVRT) as well as significantly decreased mitral valve E wave velocity and E/A ratio [49]. Our study was consistent with this having found a significantly decreased E velocity, E/A ratio and prolonged IVRT in MFS mice. The lower E velocity and prolonged IVRT in MFS mice are indicative of diastolic dysfunction with impaired ventricular relaxation [57]. A recent report determined that dilated cardiomyopathy in MFS mice is a primary manifestation caused by ECM-induced abnormal mechano-signaling by cardiomyocytes [58]. These findings suggest that the abnormal extracellular connective tissue matrix of the myocardium may also contribute to the LV dysfunction in patients with MFS. However, a recent study demonstrated an early onset of hypertrophic cardiac remodeling and dilatation of the LV in *Fbn1*^*C1039G/+*^ mice was not associated with the increase of interstitial fibrosis [59]. Since aortic dilation and stiffness also occurred in both 6-and 12-month groups, it is not clear whether the increased LV muscle mass and abnormal diastolic function in our study are due to the abnormal afterload caused by increased aortic stiffness or by abnormal myocardial connective tissue or a combination of both. A recent study also indicated that 14 month old *Fbn1*^*C1039G/+*^ mice demonstrated mild intrinsic LV dysfunction [60]. Both extracellular matrix and molecular alterations appear to be implicated in MFS-related cardiomyopathy but further studies are required to resolve this controversy.

## Conclusions

This is the first *in vivo* MFS mouse study to use high resolution ultrasound to replicate human MFS non-invasive studies and showed a similar pattern of aortic dilation and increased PWV. This method of determining PWV in mice has the potential for longitudinal in vivo monitoring of the aorta in MFS mice. High-resolution OCT found that the estimates of three dimensional parameters (e.g. volume) using 2D echocardiography were fairly accurately measured in WT mice but echo using the Teichholz estimation tended to overestimate the volumes in MFS suggesting that ventricular remodelling occurs in this syndrome. Abnormal left ventricular systolic function and early diastolic dysfunction were also found. We also demonstrated for the first time an age-dependent increase in PWV and therefore a progressive increase aortic stiffness in MFS mice similar to the human condition.
